# The improved thalamo-cortical spiking network model of deep brain stimulation

**DOI:** 10.3389/fncom.2025.1662598

**Published:** 2025-08-13

**Authors:** AmirAli Farokhniaee, Siavash Amiri

**Affiliations:** ^1^Department of Psychology, Neuroscience & Behaviour, McMaster University, Hamilton, ON, Canada; ^2^Didab PVT Ltd Co, Tehran, Iran

**Keywords:** deep brain stimulation, beta power, synchronization, thalamo-cortical network, spiking neural networks, short-term synaptic plasticity, network model, Parkinson's disease

## 1 Introduction

Parkinson's disease (PD) is a neurodegenerative disease associated with the loss of dopamine-producing neurons in the brain and is characterized by abnormal neural firing within the cortex and basal ganglia regions of the brain. Deep Brain Stimulation (DBS) is an established therapy for PD and is used clinically to relieve motor symptoms. The majority of research to-date on the symptoms of PD and their control by DBS has focused on the altered basal ganglia function (Wiecki and Frank, 2010; Caligiore et al., 2016). However, recent studies suggest that the key neural activity changes may be mediated and/or driven by the motor cortex (Lindenbach and Bishop, 2013). There are compelling clinical reasons to consider the importance of the motor cortex in the generation of symptoms and cortical stimulation as a potential therapeutic in PD (Caligiore et al., 2016; Arbuthnott and Garcia-Munoz, 2017). This is critical in understanding the role of antidromic activation of the cortex and alterations in cortical firing patterns in the therapeutic efficacy of DBS (Anderson et al., 2018). There has been a vast investigation in understanding DBS mechanisms of action during the last two decades. Recently, a thalamo-cortical microcircuit model of PD and DBS (TCM) was developed and introduced to the computational neuroscience community interested in network models of DBS (Farokhniaee and Lowery, 2020, 2021). Since the TCM model exhibited known neurophysiological features in PD and manifested DBS cortical effects in animal and human models despite its simplicity, it received a particular attention. Therefore, in this article, we fix a typo in the original article and suggest an improved version of the model to ensure the correct integration of the noise term. We also provide parallel computing and running on graphical processing unit to increase the computational efficiency of the model, useful for simulations with a higher number of neurons.

## 2 The improved model

### 2.1 TCM model structure

Certain alterations of synaptic weights within and between thalamus and cortex in a neural mass model of thalamocortex led to elevated beta power (~13–30 Hz oscillations) in the rats motor cortex (Reis et al., 2019), a well-known neurophysiological activity in parkinsonism. On the other hand, a rat model of PD showed the exaggerated synchronized patterns of spiking neurons in addition to the exaggerated beta power (Li et al., 2012). Inspired by these studies, the spiking neuronal network of thalamocortex in PD-like conditions was introduced (Farokhniaee and Lowery, 2020) and developed as a network model of DBS, known as TCM (Farokhniaee and Lowery, 2021). The developers of TCM introduced an important underlying mechanism of action of DBS at high frequencies that is the synaptic suppression due to short term synaptic plasticity (Farokhniaee and McIntyre, 2019) so that TCM served as a biophysically-realistic network model of DBS. As such, TCM exhibited known network effects of DBS that includes elevated beta power, exaggerated synchronized pattern of neuronal spikes, and formation of neuronal clusters such as excited and inhibited ones and optimized intensity of DBS-induced electric field to deliver the most suppression of the elevated beta power.

TCM contains 540 subthreshold noise-driven spiking neurons that obey Izhikevich neuronal dynamics (Izhikevich, 2003) inherently, connected via Tsodyks-Markram synapses (Tsodyks and Markram, 1997). The excitatory populations in the primary motor cortex were distributed into three layers: supragranular or surface (S, 100 neurons), granular or middle (M, 100 neurons), and infragranular or deep (D, 100 neurons) with a shared population of cortical inhibitory neurons (CI, 100 neurons). Thalamo-cortical relay nucleus (TCR, 100 neurons) and thalamic reticular nucleus (TRN, 40 neurons) form the excitatory and inhibitory populations of thalamus, respectively. The distribution of neurons in each substructure of the model along with complete neuron and synapse parameters are already presented in (Farokhniaee and Lowery, 2021).

The network dynamics of the model are described by the following set of equations (Equation 1). In the original paper (see Equation 2 in Farokhniaee and Lowery, 2021), *i* and *j* indices were also placed in the summation that includes postsynaptic currents (PSCs) and here we provide the correct formula where the summation must be over *i'* and *j'* only to obey the correct mathematical logic:


(1) v˙ij=0.04vij2+5vij−uij+140+Iij+∑j'=16∑i'=1Njωi'j',PijSCi'j'

$$\begin{array}{r} \left(t-{\text{Δ}}_{j,j'}\right)+\sum_{k,t_{k}}u_{jk}\delta (t-t_{k})+\xi \left(t\right)+I_{dbs}\delta_{j3}\\ {\dot{u}}_{ij}=a_{ij}(b_{ij}v_{ij}-u_{ij})\\ \text{if }v_{ij}\geq v_{p_{ij}}+\zeta (t)\;\text{then}\;v_{ij}\to c_{ij}\;\text{and}\;u_{ij}\to u_{ij}+d_{ij} \end{array}$$

For each structure of the TCM model*, j* = *1, 2, 3, 4, 5, 6* corresponds to the structures S, M, D, CI, TRN and TCR, respectively. For each neuron *i* = *1, 2, …, N*_*j*_ in structure *j* (where *N*_*j*_ is the total number of neurons in layer *j*) *v*_*ij*_ is the membrane voltage and *u*_*ij*_ represents the membrane recovery variable, where *a*_*ij*_*, b*_*ij*_*, c*_*ij*_, and *d*_*ij*_ are each neuron parameters, with random changes that provide non-identical neurons in the network. *I*_*ij*_ are the bias currents with ξ*(t)* and ζ*(t)* as white gaussian noises. *I*_*dbs*_ is the DBS-induced intracellular transmembrane current that is added only to layer D (*j* = 3) by means of the Kronecker delta function. The synaptic connections deliver the PSCs to the neurons by means of a weighting matrix with ω_*ij*_ elements. $\underset{k,t_{k}}{\sum }u_{jk}\delta \left(t-t_{k}\right)\;$presents poissonian background noise, with inputs occurring at time *t*_*k*_ in each structure. The PSCs are the solutions *I* in Tsodyks-Markram dynamics given by Equation 2.


(2) $$\begin{array}{l} \dot{u}=-\frac{\;u}{\tau_{f}}+U\left(1-u^{-}\right)\delta \left(t-t_{s}-\text{Δ}\right) \end{array}$$

$$\begin{array}{r} ẋ=-\frac{1-\;x}{\tau_{d}}-u^{+}x^{-}\delta \left(t-t_{s}-\text{Δ}\right)\\ İ=-\frac{\;I}{\tau_{s}}+Au^{+}x^{-}\delta \left(t-t_{s}-\text{Δ}\right), \end{array}$$

where *x* represents the fraction of available neurotransmitters after synaptic transmission and *u* is the fraction of available neurotransmitter resources ready to be used. *t*_*s*_ is the spike time, δ is the Dirac delta function, *U* is the increment of *u* produced by an incoming spike, τ_*f*_, τ_*d*_, .and τ_*s*_ are the decay (or recovery) time constant of variable *u, x* and *I*, respectively. *A* is the absolute synaptic response.

### 2.2 Simulation

In this opinion article we suggest a computational code that ensures embedment of all to all random connectivity by completely rewriting the code in a matrix-based computation at each time-step, that also increased the speed and efficiency of the algorithm (see for example updateTimeStep.m file in the model repository). We also identified a numerical issue in integrating the noise term in the original article computer code that is now edited and improvised by removing the multiplication of the noise term with each time step, though it was not creating a significant issue that lead to false results in the original study since this issue manifests during longer run times than the published results. As another model enhancement, when simulating the model using CPU, the parallel algorithm is utilized in updating each timestep through the whole network (multicore and multithread computing). In addition to solving by CPU power, we developed this parallelization on graphical processing unit (GPU, available only on Windows machine), useful in reducing the run time for simulations that include a higher number of neurons and populations. The simulations that run on GPU show their strength when the number of neurons is much higher than a several hundreds. Setting a desirable subpopulation to 0 will lead to deactivation of that subpopulation from the whole network.

**Figure 1:**
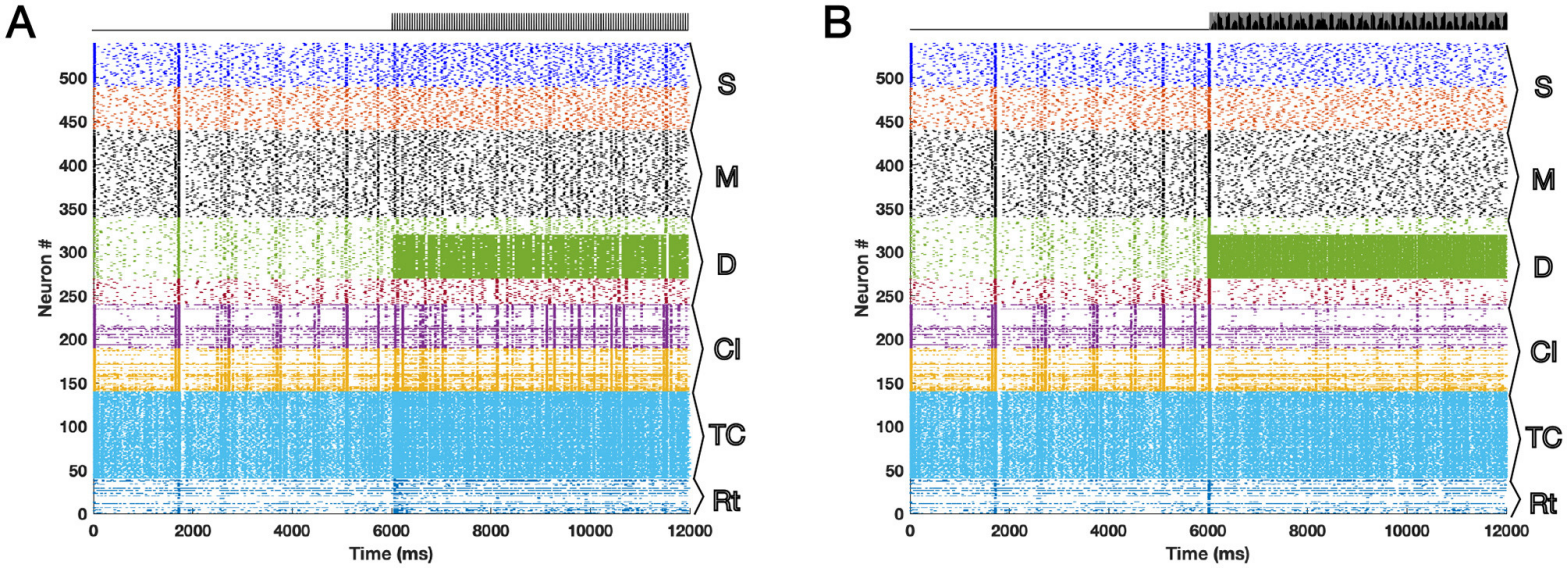
The raster plots of the TCM network for 12 s are presented here with the DBS onset at second 6 for different DBS frequencies **(A)** 20 Hz and **(B)** 130 Hz. The desynchronization effect of 130 Hz DBS in contrast to 20 Hz DBS is visible. The effect takes place via propagation of the signals in the network through synaptic connections.

The results of the model simulations for 12 seconds are shown in Figure 1 as raster plots. We ran the model on both Windows and Macintosh machines with success. We neglected the poissonian background noise in running the simulations. The white Gaussian noise had the mean of zero and the standard deviation of 0.5. The threshold noise was set to have the mean of zero and the standard deviation of 0.1. Figure 1A shows the effect of DBS at 20 Hz where it is seen that the synchronized activity is present after the DBS onset (second 6) whereas Figure 1B is the same network but during 130 Hz DBS in which the synchronized activity of the populations is diminished.

## 3 Discussion

In this article we provided an opinion about the already published network model of DBS, called TCM that has shown promising network effects in both animal (rat and monkey) and human models during rest and no visual stimuli. We suggested further developments of the TCM model by fixing a typo of the equations in the original paper, improving integration of the noise term in the original model computer code and ensuring all-to-all random network connections. The typo in the formula of the original study did not affect the original simulation results and was present only in the main text and not in the computational code of the model. We also enhanced the model computational efficiency, i.e., higher simulation speed, and inclusion of parallel computing and GPU power. DBS at high frequencies (>100 Hz) particularly around 130 Hz has been used clinically for more than 30 years in alleviating PD symptoms. Its neurophysiological effects are known mostly with suppression of exaggerated beta power and desynchronization of neuronal network firing of action potentials that is elevated during Parkinsonism. Application of DBS at 130 Hz on TCM resulted in desynchronization of the spikes as shown in the raster plot, Figure 1B, in contrast to 20 Hz DBS, Figure 1A. One of the downfalls of low frequency DBS is the resonance effects and production of unwanted harmonics. These effects plus the strengths of the TCM model in explaining clinical implications of DBS at high frequencies have been well discussed already in the original article (Farokhniaee and Lowery, 2021). TCM has limitations that could be addressed in the future studies such as inclusion of naturalistic stimuli to thalamus and simulate a suprathreshold dynamics for non-rest situations. The synaptic model of the network can be substituted easily with other known models of synaptic activity. Inclusion of long-term synaptic mechanisms such as spike-timing dependent plasticity (STDP) is another aspect that can be addressed in future studies. We suggest researchers and readers to use the improvised model presented here as the platform for future developments of DBS network model. The computational code is available online (see Data Availability Statement).

## Data Availability

The improved model presented in this study can be found in the TCM_DBS repository: https://github.com/aafarokh/TCM_DBS.
